# Chromogenic Mechanisms of Colorimetric Sensors Based on Gold Nanoparticles

**DOI:** 10.3390/bios13080801

**Published:** 2023-08-10

**Authors:** Yanyun Cui, Jun Zhao, Huidan Li

**Affiliations:** College of Chemistry and Materials Engineering, Beijing Technology and Business University, Beijing 100048, China; zhaojun@st.btbu.edu.cn (J.Z.); lihuidan@st.btbu.edu.cn (H.L.)

**Keywords:** colorimetric sensing, gold nanoparticles, enzyme-like activity, aggregation, surface modification, deposition, etching

## Abstract

The colorimetric signal readout method is widely used in visualized analyses for its advantages, including visualization of test results, simple and fast operations, low detection cost and fast response time. Gold nanoparticles (Au NPs), which not only exhibit enzyme-like activity but also have the advantages of tunable localized surface plasmon resonance (LSPR), high stability, good biocompatibility and easily modified properties, provide excellent platforms for the construction of colorimetric sensors. They are widely used in environmental monitoring, biomedicine, the food industry and other fields. This review focuses on the chromogenic mechanisms of colorimetric sensors based on Au NPs adopting two different sensing strategies and summarizes significant advances in Au NP-based colorimetric sensing with enzyme-like activity and tunable LSPR characteristics. In addition, the sensing strategies based on the LSPR properties of Au NPs are classified into four modulation methods: aggregation, surface modification, deposition and etching, and the current status of visual detection of various analytes is discussed. Finally, the review further discusses the limitations of current Au NP-based detection strategies and the promising prospects of Au NPs as colorimetric sensors, guiding the design of novel colorimetric sensors.

## 1. Introduction

The colorimetric signal readout method is a signal reading method that detects the surface refractive index of structures to be measured by recording the color changes of sensor structures based on optical response signals [1]. Researchers can observe changes in the shade or type of solution color during reactions to detect the target with the naked eye or use a UV–visible absorption spectrometer or a fluorescence spectrometer to read out signal changes. In situ, non-destructive and markerless testing of samples can be performed without other complex or large instruments. The colorimetric signal readout method is widely used in visual bioanalysis for its advantages including visualization of test results, simple and fast operation, low detection cost and fast response time. The traditional colorimetric signal readout method is very effective for the detection of certain specific substances. However, the sensitivity is limited in practice, due to the complex detection environment and the diversity of samples. There are still some design challenges for the colorimetric signal readout of detection platforms. With the development of nanoscience and technology, colorimetric biosensors are gradually tending to miniaturize and expanding towards in vivo monitoring, online monitoring, real-time monitoring and portable monitoring. Researchers are focused on designing a colorimetric sensor that can detect multiple targets simultaneously to accomplish multi-functional and multi-purpose detection and gradually building a low-cost, high-sensitivity and high-specificity detection platform [2,3,4].

In recent years, more and more metallic nanomaterials have been used for the construction of colorimetric sensors, such as gold/silver nanospheres (Au/Ag NSPs), gold nanorods (Au NRs), gold/silver nanotriangles (Au/Ag NPLs), gold nano-bipyramids (Au NBPs), gold nanostars (Au NSs), copper nanoclusters (Cu NCs) and iron tetroxide nanoparticles (Fe_3_O_4_), which have been widely used for the detection and analysis of life-related substances and chemical contaminants due to their unique physical and chemical properties [5,6,7,8,9,10]. Among them, gold nanoparticles (Au NPs) are widely used in the construction of colorimetric sensors because of their tunable localized surface plasmon resonance (LSPR), high stability, biocompatibility and easy modification [11]. It is shown that the LSPR properties of Au NPs are closely related to their morphology, size and composition. For example, Au NSPs produce a single sharp peak in the visible region, while other anisotropic nanoparticles, such as Au NRs, have two typical absorption peaks—the transverse plasmon resonance peak and the longitudinal plasmon resonance peak—due to their different aspect ratios. If the shape of Au NPs is changed, the aspect ratio of the Au NPs’ structure, or the sharpness of the multi-branched structure, changes the peak position of the Au NPs, which can be detected by UV–vis spectroscopy to reflect the size and morphology of the Au NPs by detecting their absorption peaks. Based on the LSPR properties of Au NPs, Au NPs with different sizes and morphologies absorb different light and, thus, can show different colors in solution. In addition, Au NPs have good catalytic activity and are an important nano-enzyme. Based on the above properties, colorimetric sensors constructed by Au NPs can achieve early screening and detection of many diseases and physiological conditions [12]. Researchers have synthesized Au NPs with various sizes and shapes, such as spheres, rods, cubes, prisms, stars, cages, polygons and many others, using chemical synthesis, self-assembly and molecular fabrication techniques [13,14,15,16,17,18,19]. In recent years, many reviews on colorimetric Au NP-based sensors have been published. Most of them cover the synthesis, functionalization and characterization of Au NPs and related applications [20,21,22,23]. However, few carefully categorized discussions relate to their colorimetric mechanisms specifically in the scientific literature. Furthermore, with the rapid progress in this field in recent years, it is necessary to update the information.

In this review, the current status of Au NPs based on different color development principles for visual detection of various analytes is discussed and two strategies for the construction of Au NP colorimetric sensors are summarized, including simulation of enzyme catalytic activity and Au NPs’ own LSPR phenomenon, and recent advances in Au NP colorimetric sensing in recent years are presented. Finally, we highlight the current challenges and future perspectives and summarize the possibilities and priorities for further development of colorimetric sensing methods based on Au NPs in the food industry, environmental detection and clinical medicine, providing valid ideas for fields’ future development.

## 2. Sensing Strategy Based on the Activity of Au NP-Like Enzymes

Nano-enzymes, a class of nanomaterials with catalytic activity similar to that of natural enzymes, have received much attention in recent years [24,25]. Nano-enzymes have the “dual identity” of enzymes and nanomaterials, making them a bifunctional or multifunctional molecule [26]. However, nanomaterials with different physicochemical properties exhibit different catalytic activities to those of natural enzymes, showing the complexity and diversity of nano-enzymes in enzyme-like catalysis. With the continuous enrichment of new nano-enzymes, more than 1000 nano-enzymes of different materials have been reported, expanding from metal monoatomic nanomaterials to hybridized nanomaterials such as those produced by metal monoatomic carbon doping [27]. Nano-enzymes are diverse and have various compositional structures. This section mainly reviews single-atom gold nano-enzymes based on Au NPs and composite nano-enzymes formed by hybridization of other substances with gold. It discusses colorimetric sensing strategies based on the modulation of the catalytic activity of nano-enzymes.

### 2.1. Gold Nano-Enzymes

**Figure 1:**
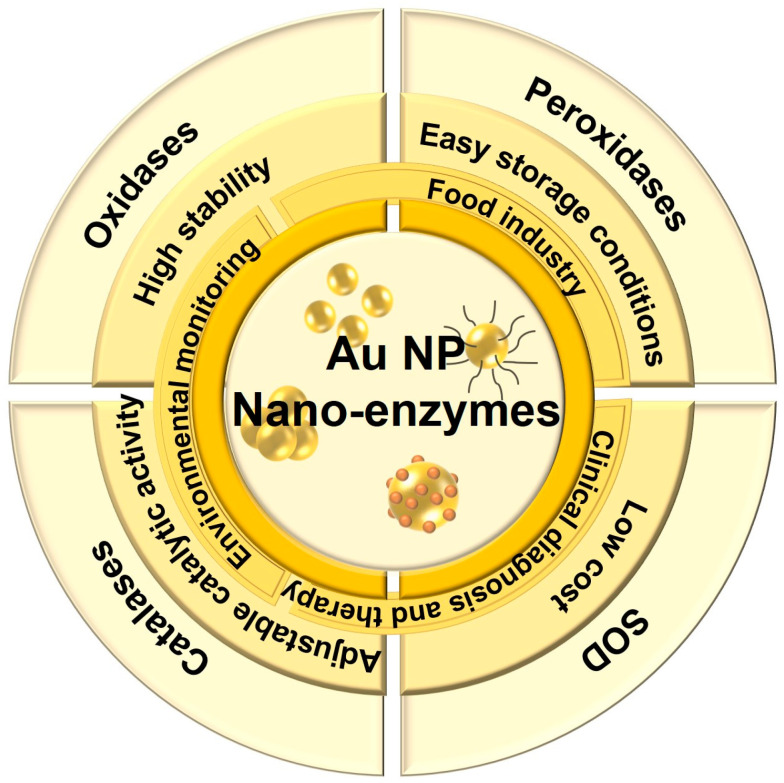
Characterization and application of Au NP-based enzyme-like activities.

Noble metal-based nanomaterials have been widely used in biosensing applications, especially Au NPs. Compared with natural enzymes, Au NPs not only exhibit adjustable catalytic activity of mimetic enzymes, such as oxidases, peroxidases, superoxide dismutases (SOD) and catalases [28,29,30,31,32,33,34], but also have advantages of high stability, easy storage conditions and low cost, making them widely used in environmental monitoring, the food industry, clinical diagnosis and therapy and other fields (Figure 1) [35,36,37,38,39,40,41].

**Figure 2:**
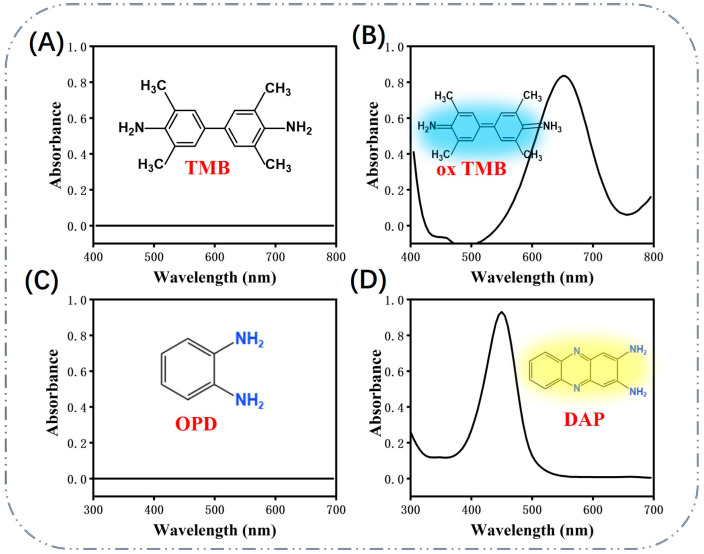
UV–vis absorbance spectra of (**A**) TMB, (**B**) ox TMB, (**C**) OPD, (**D**) DAP.

Scientists have used the physicochemical properties of Au NPs, such as nano-enzymatic properties and color visualization, to construct many novel biosensing systems for the detection of a wide range of substances. Au NPs can catalyze the conversion of targets into oxides, which is similar to the activity of natural enzymes. Di et al. developed a photoelectrochemical glucose sensor [42]. They used Au NPs as glucose oxidase mimics to catalyze glucose oxidation in the presence of oxygen while consuming oxygen, resulting in a decrease in the cathodic photocurrent. The linear response range of glucose concentration was 1.0 μM~1.0 mM with a detection limit of 0.46 μM using this sensor. Long et al. investigated the enzymatic activity of intrinsic glucose oxidase of single Au NPs and Ag–Au nanohybrids by single Au NP collisional electrochemical measurements [43]. They provide a new approach for the accurate characterization of the intrinsic catalytic activity of nano-enzymes, which provides further insights into the design of efficient nanomaterial catalysts. Moreover, this activity of Au NPs has already demonstrated strong superiority in the construction of biosensors as well as other biotechnologies. Au NPs also act as SOD. Yi et al. reported the temporal evolution of SOD aggregates associated with amyotrophic lateral sclerosis pathology using a highly sensitive and colorimetric method based on Au NPs [44]. This newly designed Au NP-based colorimetric detection system they proposed allows for the sensitive visualization and characterization of the structural evolution of protein aggregates using the protein’s own monomer-conjugated Au NPs. The elimination of O_2_•^−^ is achieved by decomposition of O_2_•^−^ into H_2_O_2_ and O_2_. The catalase activity of Au NPs was similar to that of SOD, and the catalase activity of Au NPs tended to increase under alkaline conditions and decrease under acidic conditions [45]. The peroxidase mimic activity of Au NPs is the preferred method for the development of sensors and can catalyze various substrates such as tetramethylbenzidine (TMB), 2,2-azino-bis (3-ethylbenzothiazoline-6-sulfonic acid) diammonium salt and o-phenylenediamine (OPD). Therefore, Au NPs have been used as a signal sensor capable of generating colorimetric signals. The mechanism of Au NPs as peroxidases is based on the formation of peroxide bonds and two hydroxyl radicals on the surface of gold nano-enzymes. The radicals produced by Au NPs are stabilized by electron exchange interactions, which also contribute to the catalytic ability of gold nano-enzymes. As shown in Figure 2A,B, TMB has no UV absorption peak and is transformed into ox TMB after the action of the enzyme, with a strong absorption peak at 650 nm. It is favored by researchers for its promising stability because there is no need to avoid light after coloration or mutagenic effects. OPD is also a widely studied chromogenic substrate that has no UV–vis absorption in the range of 300–700 nm (Figure 2C). Au^3+^ promotes the loss of an electron from OPD to produce an OPD radical, which further loses another electron to form the intermediate quinone imine. The two quinone imine molecules then synthesize the final product, 2,3-diaminophenazine (DAP), which has a characteristic absorption peak at 450 nm and appears yellow in color (Figure 2D). In this electron transfer process, Au^3+^ gains electrons to produce Au^+^, which in turn is oxidized by hydroxyl radicals to give Au^3+^ [46].

**Figure 3:**
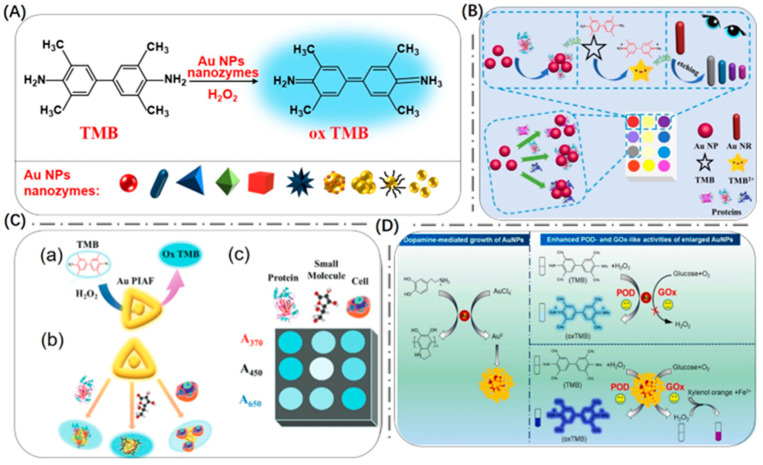
(**A**) The color reaction principle of TMB based on Au NP nano-enzymes and H_2_O_2_. (**B**) Schematic illustration of the multicolor sensor array. Reproduced with permission from [47]. Copyright 2020, Elsevier. (**C**) Schematic of the nano-enzyme sensor array based on Au PIAF for detecting multiple substances. (**a**): Schematic diagram of Au PIAF-catalysed TMB. (**b**): Schematic diagram of the binding of Au PIAF and different targets. (**c**): Schematic diagram of the detection of the targets using the absorbance at different absorption wavelengths as the sensor element. Reproduced with permission from [48]. Copyright 2021, Royal Society of Chemistry. (**D**) Schematic illustration of dopamine-mediated growth of Au NSPs enhancing their dual-enzyme mimetic activity. Reproduced with permission from [49]. Copyright 2023, Elsevier.

In recent years, most researchers have constructed colorimetric sensors based on Au NPs with peroxidase-like catalytic activity for the oxidation of TMB and achieved efficient detection of the target by oxidizing TMB to produce color changes (Figure 3). The detection principle of these sensors is shown in Figure 3A, where TMB is converted into ox TMB in the presence of Au NP nano-enzymes and H_2_O_2_. As shown in Figure 3B, Wang et al. used the peroxidase-like activity of Au NSPs to catalyze the conversion of TMB to TMB^2+^. At the same time, the aggregation and dispersion of Au NSPs were used to regulate their catalytic activity and realize biosensing [47]. 

In order to better exploit the catalytic activity of Au NPs as nano-enzymes, researchers can also modulate their size and morphology by precisely regulating their strategies. As shown in Figure 3C, Wang et al. proposed a sensor array for recognition of multiple targets. They investigated the catalytic properties of Au NPs and synthesized particle-in-a-frame nanomaterials (Au PIAF) with internal nano-gaps using a controlled replacement method of Ag nano-trigonal prisms [48]. Compared with Au NSPs and gold nano-prisms, Au PIAF had larger specific surface area due to the internal nano-gaps and gold particles in the framework, which showed good catalytic performance and good stability and might have displayed promising application prospects in catalytic sensing. Au PIAF were used to simulate peroxidase-catalyzed substrate TMB reactions, and the colorless TMB became a blue product in the presence of H_2_O_2_. The presence of the target inhibited the reaction between TMB and Au PIAF. Different proteins had different inhibitory abilities in the system, and each protein varied across the three wavelengths because each protein had a different adsorption capacity and a different coverage area of the catalytic site. And the catalytic performance of Au PIAF was affected, resulting in changes in absorbance at different absorption wavelengths and sensor elements. The system successfully achieved the identification of seven proteins in water samples and complex serum systems. As shown in Figure 3D, Guo et al. found that dopamine-mediated production of Au NSPs significantly accelerated the peroxidase- and glucose oxidase-like activities of Au NSPs [49]. They used dopamine as a reducing agent to reduce Au^3+^ salts to Au^0^, which was deposited on the surface of Au NSPs, resulting in the growth of Au NSPs into highly branched Au super-granules of larger size. These dendritic Au super-particles could significantly accelerate their enzyme-like activity, which would in turn enable sensitive and selective colorimetric detection of dopamine and glucose, respectively.

### 2.2. Composite Nano-Enzymes

In addition to the size and morphology of nanoparticles, the internal structural composition of the surface functional layer or nanomaterial also plays a decisive role in regulating nano-enzyme activity. Surface modification and hybridization are important approaches for regulating the internal structure and composition of nano-enzymes.

#### 2.2.1. Surface Modification 

**Figure 4:**
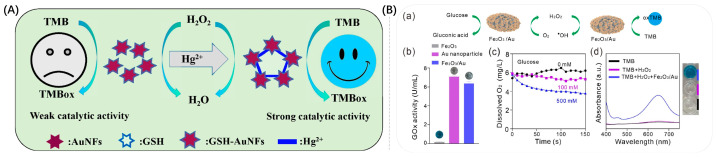
(**A**) Schematic diagram of GSH-Au NF aggregation and activated peroxidase induced by Hg^2+^. Reproduced with permission from [53]. Copyright 2022, Elsevier. (**B**) (**a**): Schematic description of Fe_2_O_3_/Au hybrid nano-enzyme for glucose consumption and •OH generation. (**b**): Comparison of GOx activity of Fe_2_O_3_, Au NPs and Fe_2_O_3_/Au. (**c**): The changes in dissolved O_2_ over time are induced by Fe_2_O_3_/Au in the presence of glucose with different concentrations (0, 100 and 500 mM). (**d**): absorption of TMB incubating with H_2_O_2_ and Fe_2_O_3_/Au. Reproduced with permission from [56]. Copyright 2023, Elsevier.

By coating DNA corona (Au NPs @ DNA) on Au NPs, Shen’s group merged nano-enzymes and DNA enzymes into a novel artificial enzyme with a catalytic efficiency that is 5-fold higher than that of Au NP nano-enzymes, 10-fold higher than that of other nano-enzymes, and significantly higher than that of most nano-enzymes [50]. Feng et al. dutifully wrapped β-lactoglobulin amyloid fibrils (BLGF) against Au NPs [51]. Compared with natural β-lactoglobulin (BLG)-stabilized Au NPs, BLGF-Au NPs showed a 3.2-fold enhancement in fluorescence and a significant increase in redshift by 42 nm. According to the Michaelis–Menten equation, the kinetic parameters of the BLGF-Au nano-enzymes showed a lower value of Km for TMB (66 μmol/L) and a high vmax value (3.74 × 10^−8^ M/s) for H_2_O_2_, which is comparable to other artificial nano-enzymes and natural peroxidases. Cao et al. proposed a simple, low-cost and easy-to-use sensing method based on the convertible peroxidase mimetic activity of Au NSPs, which catalyzes the oxidation of TMB for protease determination [52]. The Au NSPs’ surface was modified with casein to show dual functionality. In the presence of protease, the enzyme binds and catalyzes the degradation of the Au NSPs’ surface coating, leading to the restoration of peroxidase mimetic activity. This is shown visually in the development of a blue product or spectroscopically as an increase in absorbance at 370 and 650 nm. This mechanism allowed the detection of 44 ng/mL of protease within 90 min. The sensing method has also been used for the detection of spiked proteases in ultraheat-treated milk and synthetic human urine samples with detection limits of 490 and 176 ng/mL, respectively, and shows great potential in clinical diagnostics, food safety and quality control. As shown in Figure 4A, Bai et al. found that glutathione-modified gold nanoflowers (GSH-Au NFs) had activated peroxidase mimetic activity and developed an ultrasensitive colorimetric detection method for Hg^2+^ [53]. After the addition of Hg^2+^, the GSH-Au NFs would rapidly aggregate due to the interaction of Hg^2+^ with the carboxyl and amino groups of GSH. Due to the aggregation, they showed high peroxidase-like catalytic activity, which could catalyze TMB in the presence of H_2_O_2_, leading to subsequent color reactions. With a linear range of 10–300 nM and detection limits as low as 3.9 nM, the accuracy of the method has been demonstrated in water samples and showed great potential for environmental monitoring. Cheng et al. used polymer–ligand hydrogen bonding to improve Au NSP-based nano-enzymes and enhanced their mimetic enzymatic activities. They prepared poly(N-2-hydroxypropylmethacrylamide)-capped Au NSPs (PHPAM @ Au NSPs). They constructed a colorimetric sensing assay for monitoring homocysteine (Hcy), based on the inhibition of the catalytic activity of PHPAM @ Au NSPs by Hcy. In this method, they relied on the enzyme-like catalysis of the nano-enzyme to oxidize TMB to oxidized TMB (ox TMB). The UV–visible absorption intensity of ox TMB showed a strong linear relationship (R^2^ = 0.998) with Hcy concentration in the range of 3.0–20.0 μM, with a detection limit of 0.4 μM [54]. Gold nanoclusters capped with papain synthesized by Ma et al. also exhibited efficient peroxidase-like activity. This illustrates that negatively charged dopamine could trigger the aggregation of positively charged gold clusters and reactive oxygen species generated during oxidation, which would lead to a significant enhancement in its catalytic activity [55]. 

#### 2.2.2. Hybridization

In order to further enhance the catalytic activity of nanomaterials mimicking enzymes, composite nano-enzymes doped with gold nanoparticles and other substances have been synthesized. It has shown that the interaction between two or more components allows nano-enzymes to exhibit novel properties and their enzyme-like activity can be regulated by changing the ratio of different components in the nanomaterials, showing surprising synergistic effects in the field of biosensors and immunoassays. For example, Zhou et al. found that Au NP/GeO_2_ nano-enzymes exhibited stronger peroxidase-like activity than single Au NPs or GeO_2_ [57]. Zhong et al. synthesized a novel H_2_O_2_ dual sensor constructed from TMB-Fe_3_O_4_@Au NPs by combining colorimetry and surface-enhanced Raman spectroscopy [58]. The linear range was from 40 μM to 5.5 mM with a detection limit of 11.1 μM in a colorimetric assay, and the sensor was successfully applied to the detection of H_2_O_2_ in plasma and milk, showing excellent performance and flexibility. As shown in Figure 4B, Zeng et al. fabricated a chitin-derived porous Fe_2_O_3_/Au hybrid nano-enzyme to synergistically treat triple-negative breast cancer [56]. Their conversion of minerals into nano-enzymes and coupling of nano-enzymes for the synergistic treatment of tumors provide insights into the rational design of nano-enzyme composites for biomedical applications. Since nanocomposites have shown good performance in bioactivity, more researchers have used this composite structure to control the catalytic activity of nano-enzymes with good results.

The number of active sites where nano-enzymes are in contact with reaction substrates directly affects the catalytic activity of nano-enzymes. The exposure of active sites to nano-enzymes and their structural similarity to the active sites of natural enzymes are improved by reducing the size of nano-enzymes, increasing their surface area and improving the surface area/volume ratio, thus enhancing the catalytic activity of nano-enzymes [25]. The larger the specific surface area of a nano-enzyme is, the higher the number of active sites and the higher the level of catalytic activity will be. Huang et al. investigated a highly active dual-nano-enzyme amplification biosensor by growing ultrafine Au NPs with high density and good dispersion in situ on metal–organic framework (MOF) nanosheets and developed a novel nano-enzyme based on ultrathin two-dimensional (2D) conductive MOF nanosheets and achieved highly sensitive detection of H_2_O_2_ in living cells [59]. The large specific surface area of MOF materials provided an abundance of active sites, and ultrafine Au NPs were recognized as highly active nano-enzymes, and these two discoveries could synergistically result in higher catalytic activity of the composite nano-enzymes. In addition, the specific surface area of the nano-enzymes could be enhanced by changing the morphology of the nano-enzymes to increase the number of active sites and then combined with the different properties of nanomaterials, not only to enhance the catalytic activity of the nano-enzymes but also to enrich their functions. For example, Asen et al. developed a novel non-enzymatic glucose sensor based on flower-like gold nanostructures and graphene oxide as a support matrix [60]. Zhao et al. successfully synthesized Au NR/metal–organic framework (Au NRs/Fe-MOF) hybrids for photo-enhanced peroxidase-like catalysis, which exhibited good stability and reproducibility in experiments [61]. Notably, nanomaterials with fluorescence properties have been widely used in imaging and detection fields, and the combination of enzyme-like activity and fluorescence properties of nanomaterials has led to more diversified functions of nano-enzymes. Sun et al. prepared a petal-like mixture of urate oxidase and hollow gold nanocages (Au NCs) as a novel dual-channel biosensing platform for the detection of uric acid (UA) [62]. The enzymatic catalysis of this mixture in the presence of UA triggered tandem catalytic and oxidative reactions leading to fluorescence quenching. The linear detection range of UA was between 0.1–10 μM and 10–300 μM with a detection limit of 20 nM. All these results indicated that the hybridized nano-enzyme materials have great potential in many fields.

## 3. Sensing Strategy Based on the LSPR Properties of Au NPs

**Figure 5:**
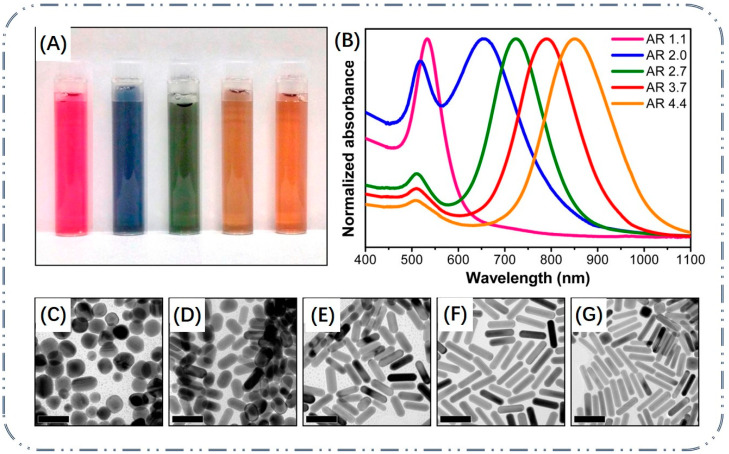
Characterization of gold nanorods with different aspect ratio: (**A**) solution photographs. (**B**) UV–vis absorbance spectra and (**C**–**G**) TEM images. The aspect ratio from left to right is 1.1, 2, 2.7, 3.7 and 4.4. Scale bars = 50 nm [65]. Copyright 2014, ACS Publications.

Most traditional colorimetric signal readout methods rely on changes to the color shade of the solution. The color change of the solution is simple and the resolution of color development is poor. The accuracy of visualization results is low for targets with similar concentrations according to the naked eye. Based on high molar extinction coefficients and the LSPR of Au NPs, a colorimetric sensor of multiple colors can be constructed as a way to improve the sensitivity of sensors. LSPR is a strong absorption of photon energy from nanoparticles when Au NPs are irradiated by incident light and the oscillation frequency of the incident light coincides with the oscillation frequency of the free electrons on the surface. LSPR peaks of Au NPs always range in the visible–NIR region. This phenomenon is closely related to nanoparticle composition, morphology, size and other factors [63]. The different sized and shaped Au NPs possess different optical properties, and their LSPR peaks can be adjusted between 500–1200 nm [64]. For example, Murphy et al. synthesized Au NRs with a size of 20–60 nm, and these nanorod solutions exhibited different colors (Figure 5A) [65]. With an increase in the aspect ratio of the Au NRs, their corresponding LSPR peaks gradually red-shifted (Figure 5B). The corresponding shape also changed from rod-shaped to spherical (Figure 5C–G). In addition, there are significant differences in the LSPR peaks of Au NPs with different shapes. For example, the LSPR peak of 50 nm Au NSPs is located at 520 nm, whereas the LSPR peaks of Au nanocubes and Au NSs with the same particle size are located at 540 nm and 700 nm, respectively [66,67]. Therefore, the position of the LSPR peaks can be adjusted by changing the shape and size of Au NPs, which is crucial for the construction of Au NP-based chemical and biological sensors [68].

**Figure 6:**
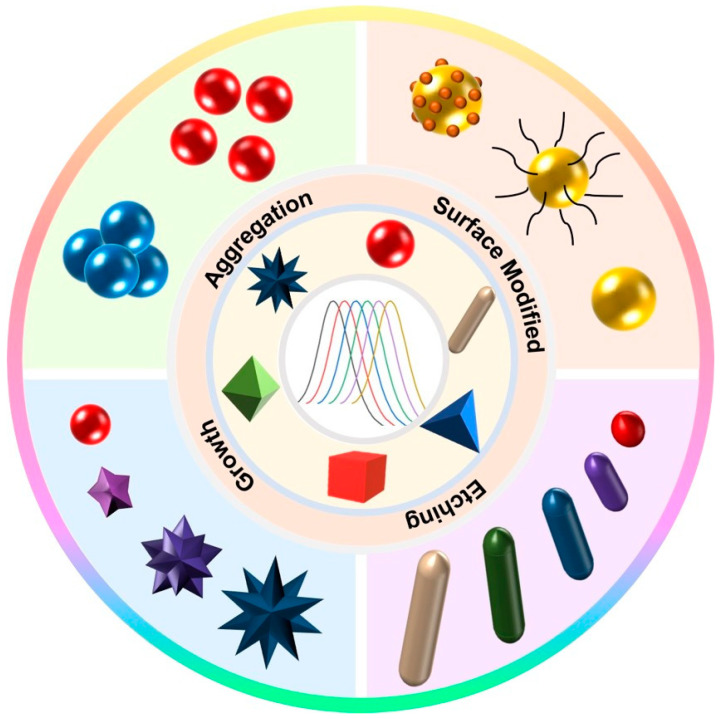
Different regulating methods of LSPR performance of Au NPs.

The LSPR of Au NPs with different shapes can be adjusted by aggregation, surface modification, deposition, etching, etc. Further, their solution color can be changed to achieve detection of the target (Figure 6). Through these methods, it is possible to change the LSPR performance of AuNPs and improve the stability and sensitivity of biosensors.

The recent progress in research on Au NPs with different actions for changing their properties and different shapes for LSPR-based applications is shown in Table 1. It includes some modifications of traditional Au NPs that are used in the presented sensors. All these methods can change the LSPR of Au NPs and realize the controllable regulation of LSPR for better detection of targets. Meanwhile, we collected data about the time of measurements for Au NP-based sensors, since the response time of LSPR-based sensors depends significantly on size, coverage, concentration and other parameters related to using Au NPs and determines the competitive potential of the proposed biosensors.

### 3.1. Aggregation

Aggregation induces changes in the morphology and size of Au NPs, resulting in shifts in their LSPR peaks, as well as changes in visible light color [80,81]. The characteristic absorption peak of LSPR for Au NSPs is at 520 nm and its solution appears bright burgundy. As the core size increases, the absorption peaks of Au NSPs usually exhibit a redshift corresponding to the size. In particular, the aggregation and dispersion of Au NSPs with diameters above 3.5 nm cause a displacement of the plasma band while the color of the solution achieves shifts from red to blue and from blue to red, respectively [82]. Due to interparticle plasmon exciton coupling, when the aggregation of Au NSPs occurs, its absorption intensity at 520 nm decreases and a new LSPR absorption peak appears in the range of 600–800 nm, and the solution color changes from burgundy to blue. The aggregation and dispersion of Au NPs can be used as a signal switch for colorimetric sensors, providing signal changes that can be judged by the naked eye without the use of large and complex optical analytical instruments, thus accomplishing simple and easy-to-use target detection.

#### 3.1.1. Bio-Specificity

The aggregation of Au NSPs can be achieved by bio-specific binding, electrostatic interactions and covalent binding.

**Figure 7:**
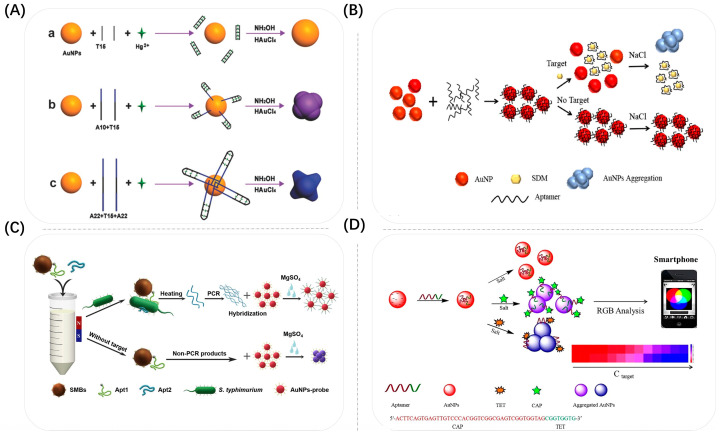
(**A**) Schematic illustration of the presented sensing strategy for the colorimetric detection of Hg^2+^ based on the growth of Au NSPs induced by different amounts of bases. The black line represents the binding domain, and the blue line represents excess bases except for the binding domain. Reproduced with permission from [84]. Copyright 2017, Wiley. (**B**) Principle of quantitative colorimetric detection of SDM based on Au NSPs aggregation with a smartphone. Reproduced with permission from [86]. Copyright 2022, Elsevier. (**C**) Schematic illustration of the proposed SMBs–Apt1 sandwich-based colorimetric sensor for detection of S. typhimurium in milk (Size not to scale). Reproduced with permission from [87]. Copyright 2020, Elsevier. (**D**) Schematic illustration of the detection of TET/CAP based on Au NSP colorimetric aptasensors. The Apt acts as a molecular switch adjusting the Au NSP aggregation. When antibiotics remove the fragment of Apt from the Au NSPs’ surface, unbalanced Au NSPs are aggregated on different scales under high-salt conditions. It thereby causes colloidal color changes, which can be detected by UV spectroscopy and Smartphone analysis, respectively. Reproduced with permission from [70]. Copyright 2020, Elsevier.

The method of using bio-specific reactions to aggregate Au NSPs is one of the more widely used binding methods to date. As early as 1997, Mirkin et al. reported a highly selective colorimetric polynucleotide detection method based on gold nanoprobes modified with sulfhydryl oligonucleotides [83]. They introduced the target oligonucleotide chain (30 nt) into the solution containing the probe, forming a nanoparticle aggregation network with red to pink to purple color that turned blue upon drying. The group designed a sensor that could detect 10 fM oligonucleotide chains without optimization, which was the most typical sensor that used bio-specific reactions to aggregate plasmonic nanoparticles, and also laid the foundation for a new generation of medical diagnostics based on colorimetry of metal nanomaterials. Subsequently, several groups realized the detection of heavy metal ions or pathogenic bacteria based on the principle of Au NSP aggregation. In recent years, sensors designed based on the aggregation-induced colorimetric effect of Au NPs using specific recognition of aptamers, antibodies and other bioreceptors have been successfully used to detect more targets. As shown in Figure 7A, Tan et al. reported a multifunctional Hg^2+^-sensitive colorimetric sensor based on aptamer target-specific binding and target-mediated growth of Au NSPs [84]. They attached specially designed aptamer DNA to the Au NSPs, and once the target Hg^2+^ was present, the aptamer would bind to the target, thus shedding it from the Au NSPs. The aptamer remains on the surface of the Au NSPs and excites the Au NSPs to grow nanostructures with different morphologies, thus allowing the solutions to shift across different colors. The group improved the sensitivity of this detection system by controlling the growth of Au NSPs by changing the length of the aptamer as well as the base species. The detection limit decreased from 9.6 × 10^−9^ M to 3 × 10^−9^ M. In addition, the aptamer (59 nt in length) could be applied to the identification of Hg^2+^ in river samples with a detection limit of 6.2 × 10^−9^ M. Ma’s group, which also detected Hg^2+^ in water, successfully constructed a novel label-free colorimetric aptamer sensor [85]. They designed a hairpin-loop DNA probe with thymidine-rich recognition termini to specifically recognize trace amounts of Hg^2+^ through a stable T–Hg^2+^–T structure. With the addition of Hg^2+^, the color of Au NSPs changed from blue to red. The limit of quantification of Hg^2+^ was 0.2 nM, and the linear range was 0.5–5.0 nM. The whole experimental testing time was about 40 min. Even real water samples containing 1 nM Hg^2+^ could be measured with this aptamer sensor with a recovery of 97–103%. In addition, the colorimetric sensor gradually developed towards portability and intelligence. As shown in Figure 7B, Li et al. established a method for the quantitative detection of sulfadimethoxine (SDM) using a Au NSP aggregation-induced colorimetric sensing smartphone [86]. The attachment of the aptamer effectively prevented the aggregation of Au NSPs in highly concentrated salt solutions. In the presence of SDM, SDM bound to the aptamer on the surface of Au NSPs with high affinity and had bio-specificity. The aptamer was shed from the surface of Au NSPs, resulting in the aggregation of Au NSPs and a color change from red to violet-blue. Under optimized conditions, the smartphone colorimetric sensing method had a high sensitivity to SDM with a detection limit of 0.023 ppm, which was lower than the maximum allowed SDM residue limit. For colorimetric detection of the target, as shown in Figure 7C, Jiang’s group proposed a method for visual detection of live Salmonella typhimurium based on Au NSPs [87]. They attached aptamer 1 to the target, which bound to aptamer 1, and then added aptamer 2, which bound to the target to form a sandwich complex. After magnetic separation, aptamer 2 was dislodged by thermal decomposition and then PCR amplification was performed. The highly stable network structure formed by hybridization between the amplified aptamer 2 and Au NSP probes protected the Au NSPs from aggregation and color change in the presence of salt. Under optimized conditions, the ratio of the absorbance intensities of Au NSPs varied regularly with decreases in the pathogenic bacteria concentration. The absorbance intensity of Salmonella typhimurium was linear in the concentration range of 3.3 × 10^1^~3.3 × 10^6^ CFU/mL. The detection limit of Salmonella typhimurium was up to 33 CFU/mL in pure culture and up to 95 CFU/mL in spiked milk. The colorimetric sensor constructed from dual aptamers can amplify the shed aptamer fragments, thus improving the sensitivity of detection. The sensor based on aptamer-Au NSPs can also perform the detection of multiple targets. As shown in Figure 7D, Wu et al. designed a novel colorimetric sensor based on coordinated control of Au NSP aggregation by single-stranded DNA fragments and selected chloramphenicol and tetracycline as model antibiotics for multi-target detection [70]. They attached designed multifunctional aptamers to the surface of Au NSPs to act as antibiotic-binding elements and molecular switches. When an antibiotic was added, the fragment specifically recognized by the aptamer could bind to the antibiotic and separate from the Au NSPs, while the non-specific fragment synergistically controlled the aggregation of Au NSPs under conditions of high salt concentration. Therefore, the different color changes of Au NSP solutions could be used as a signal readout. The sensor had good selectivity and sensitivity for the separation and detection of tetracycline and chloramphenicol with detection limits of 32.9 nM and 7.0 nM, respectively.

Antibodies are y-shaped glycoproteins that bind specifically to antigens. They have a variety of immune functions. The high affinity between antigens and antibodies provides a good strategy for constructing colorimetric sensors for bio-analyte detection. For example, Singhal et al. proposed a rapid and specific foodborne bacteria detection biosensor based on graphene oxide-coated Au NPs (GO-Au NPs). The detection mechanism of this method is based on the highly specific recognition and interaction between the antibody-Au NPs and the target, which in turn leads to a color change upon aggregation of Au NPs. The limit of detection of this colorimetric method for E. coli was 103 CFU/mL using the naked eye. The limit of detection could be reduced to 102 CFU/mL by spectrophotometry. In addition, the antibody-functionalized GO-Au NPs could specifically inactivate the bacteria after near-infrared exposure [88]. Hence, this method can enable the rapid detection and simultaneous sterilization of bacteria. Ray et al. structured a novel sensing system for rapidly diagnosing and suppressing the SARS-CoV-2 virus based on anti-spiking antibody-attached Au NPs. The sensing system allows visual detection of specific viral antigen or pseudo-SARS-CoV-2 within 5 min, and the detection limits of SARS-CoV-2 antigen and viral particles are 1 ng/mL and 1000 ng/mL [89]. 

Peptides are new bio-functional candidate molecules that have recently played an essential role in the target recognition and detection of biochemical analytes. Remarkably, peptide-Au NP-based colorimetric nanoplatforms have attracted increasing interest in pathogen detection. In a recent study, Liu et al. have developed a peptide-Au NP-based colorimetric strategy for the detection of SARS-CoV-2 primary protease. The principle of the assay is that the peptide substrate of the primary protease induces the aggregation of Au NPs, which causes a color change in the solution. In addition, the researchers used an electrochemical method for detecting the primary protease. The sensing system’s sensitivity and immunity to interference were further improved [90].

#### 3.1.2. Electrostatic Interactions

**Figure 8:**
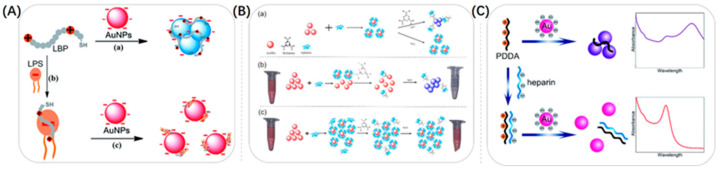
(**A**). The principle of the colorimetric sensor for the LPSs. Route (**a**): aggregation of Au NSPs induced by the LBP; route (**b**): formation of LPSs–LBP complexes; route (**c**): interaction between Au NSPs and LPSs–LBP complexes. Reproduced with permission from [91]. Copyright 2016, Royal Society of Chemistry. (**B**) (**a**): The schematic illustration of the colorimetric assay of melamine using aptamer-functionalized Au NSPs. (**b**): The effect of overuse of Au NSPs on this colorimetric assay in practical applications. (**c**): The effect of overuse of aptamers on this colorimetric assay in practical applications. Reproduced with permission from [94]. Copyright 2018, Public Library Science. (**C**) Schematic illustration of the principle for colorimetric detection of heparin. Reproduced with permission from [95]. Copyright 2019, Royal Society of Chemistry.

Another way to make Au NSPs aggregate is through electrostatic interactions. Au NSPs are charged by modification, and then the Au NSPs are made to aggregate or depolymerize with the help of the heterogeneous charges present in solution. As shown in Figure 8A, Lei et al. developed a new label-free colorimetric method for the detection of lipopolysaccharides (LPSs) based on lipopolysaccharide-binding peptide (LBP) and unmodified Au NSPs [91]. They modified the surface of Au NSPs with anions and then used a cationic LBP probe to cause them to aggregate, and the color of the solution changed from red to purple. In the presence of LPSs, the specific binding between LPSs and LBP led to the formation of LPSs–LBP complexes, which subsequently prevented the aggregation of unmodified Au NSPs. This led to Au NSPs exhibiting significant differences in their color and absorption spectra. The method was sensitive and selective for determining polysaccharides in the range of 10–1000 nM with a detection limit of 2.0 nM. Li et al. used a similar approach to inhibit the aggregation of Au NSPs [92]. They developed a simple colorimetric probe for the rapid and highly sensitive detection of malathion. A certain amount of NaOH caused Au NSPs to stabilize in a citrate environment to aggregate electrostatically, and the color of the Au NSP solution changed from burgundy to gray. The malathion was easily hydrolyzed in a strongly alkaline environment (pH > 9), generating a large number of negative charges, causing the aggregated Au NPs to become dispersed and the color of the solution to change from gray to burgundy. Using a similar approach, Lei et al. developed a Tl^+^ label-free colorimetric sensing scheme based on Au NSPs [71]. Unlike the above-mentioned groups, Yu et al. established a dual-mode detection method for organophosphate pesticides (OPs) based on a fluorescent–colorimetric sensor using the aggregation of Au NSPs [93]. Indoxyl acetate (IDA) was hydrolyzed by esterases into indophenol. Indophenol changed the LSPR signal of gold nanoparticles and led to their aggregation, which eventually changed the solution’s color from red to blue. OPs could inhibit the production of indophenol, so the LSPR signal decreased and the color change of the solution gradually diminished with an increase in OP concentration. As shown in Figure 8B, Hu et al. exploited the electrostatic interactions for the detection of melamine [94]. When melamine was present, the stable structure consisting of Au NSPs and an aptamer was disrupted and the aptamer was shed from the surface of Au NSPs, leading to the aggregation of Au NSPs. As a result, the color of Au NSPs changed from burgundy to blue during the aggregation process.

Making Au NSPs positively charged can be equally effective for detection. As shown in Figure 8C, Ma et al. reported a highly sensitive colorimetric sensor for the detection of heparin based on the anti-aggregation effect of a poly diallyl dimethyl ammonium chloride (PDDA)-Au NSPs colloidal system [95]. In the presence of positively charged PDDA, the system became electrically neutralized and Au NSPs tended to aggregate. While heparin had a high negative charge density, it could form stable complexes by strong binding with PDDA through electrostatic interactions, effectively inhibiting the aggregation of Au NSPs. The color change of the solution could be quickly distinguished by the naked eye. The method was highly sensitive and selective and has been successfully applied to the determination of human serum samples. Zhang’s group found that the positively charged Au NSPs redisperse when As^3+^-aptamers reached a certain high concentration [96]. They established a dual-mode colorimetric sensor for the determination of As^3+^ by optimizing the concentration of As^3+^-aptamers, which led to the aggregation and redispersion of positively charged Au NSPs.

#### 3.1.3. Covalent Bonding

Covalent bonding can also cause Au NSPs to aggregate. Through appropriate use of covalent bonding, researchers can simply achieve the goal of inducing or preventing the aggregation of Au NSPs. The modification of 11-mercapto-undecanoic acid on the surface of Au NSPs was proposed by Kim et al. in 2001 [97]. The alkanoic acid were bound to Pb^2+^, Hg^2+^ and Cd^2+^, so when these heavy metal ions were present in water, the Au NSPs would aggregate and change the solution from red to blue. Lin et al. used a similar approach to detect K^+^ [98]. Conversely, functionalized modification of Au NSPs by covalent binding, thereby preventing Au NSPs from aggregating, could likewise enable colorimetric detection of the target. Huang et al. presented a colorimetric bacterial detection strategy based on 4-mercaptophenylboronic acid (4-MPBA)-functionalized Au NSPs [69]. 4-MPBA-Au NSPs were immobilized on the bacterial cell surface by covalent bonding, which hindered NaCl-induced aggregation of 4-MPBA-Au NSPs. Color changes caused by Au NSP aggregation could be observed with the naked eye and quantified by UV–visible absorption spectra or Red–Green–Blue (RGB) analysis based on a digital camera for quantification. Tang et al. used a similar method for the detection of Hg^2+^ [99]. 

### 3.2. Surface Modification

**Figure 9:**
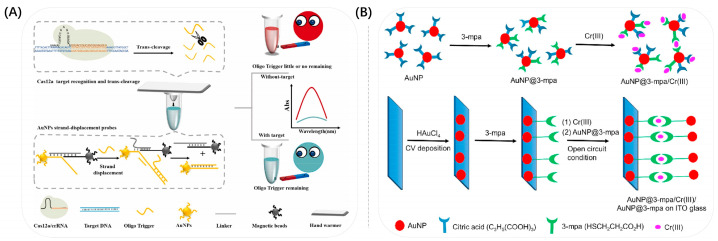
(**A**) Schematic illustration of CRISPR-Cas12a coupled with gold nanoparticle strand displacement probe for rapid and sensitive visual SARS-CoV-2 detection. Reproduced with permission from [100]. Copyright 2023, Elsevier. (**B**) Schematic representation of the preparation of sensing systems for colorimetric and electrochemical detections of chromium ions in water. Reproduced with permission from [105]. Copyright 2021, Elsevier.

Au NSPs are popular among researchers because of their excellent chemical inertness, non-toxicity and good biocompatibility. By modifying functional groups on the surface of Au NSPs, researchers can bind substances such as DNA, antibodies, peptides, proteins or small molecules to detect different target assays. Au NPs have a wide range of applications in sensing as sample pretreatment carriers, signal recognition elements or signal readout probes. While surface chemistry is the most crucial tool for nanoparticle functionalization, especially in biochemical analysis, surface modification strategies are crucial for nanoparticle-mediated analytical processes. Researchers modified thiols on the surface of Au NPs to improve the stability of the nanomaterials and also used Au–S bonds to modify DNA on Au NPs. As shown in Figure 9A, Pei et al. modified ssDNA by Au–S on Au NPs, and they combined Au NPs with magnetic beads (MBs) to form a sandwich structure to design a colorimetric sensor for nucleic acid detection [100]. Once the target activated CRISPR/Cas12a, the oligo trigger chain connecting the Au NPs to the magnetic beads was cleaved in trans and the sandwich structure was disrupted. The originally pink solution became colorless. The colorimetric sensor allowed qualitative detection by the naked eye alone and quantitative detection by UV–vis absorption spectroscopy. In addition, low-cost warmers could be used as an incubator for visual detection, enabling instrument-free, visual detection of SARS-CoV-2 within 20 min. Wang et al. established a simple colorimetric analysis method using unmodified Au NSPs and magnetic 3D DNA walkers to detect miRNA-155 down to 16.7 fM [101]. With this platform, hundreds of substrate ssDNA (DNA tracks) and dozens of blocked walking strands were attached to Au–Fe_3_O_4_ nanocomposites as magnetic 3D DNA walkers. The target activated a DNA walker driven by a DNAzyme cleavage mechanism that produced a large number of single-stranded products. After magnetic separation, the resulting displaced ssDNA was incubated with Au NSPs to prevent aggregation of unmodified nanoparticles in high-salt solutions. On this basis, the concentration of miRNA-155 could be visualized by a color change from gray-black to purple by the composite probe and quantified by UV–vis absorption spectroscopy. 

Shahdordizadeh et al. chose to ligate Au NSPs to double-stranded DNA consisting of an aptamer and its complementary strand for the detection of Pb^2+^ [102]. In the absence of Pb^2+^, the dsDNA on Au NPSs prevented the aggregation of Au NSPs at high salt concentrations. In contrast, in the presence of Pb^2+^, the aptamer bound to Pb^2+^ and the complementary strand was released and digested by exonuclease I. In contrast, Xu et al. constructed a colorimetric method for the determination of kanamycin by hybridization chain reaction-assisted signal amplification using Au NSPs as signal conversion elements [103]. The presence of kanamycin disrupted the structure of hairpin DNA, and the addition of NaCl solution caused the Au NSPs to aggregate, thus producing a shift from red to blue. Kou et al. synthesized 7-hydroxycarbonyl-2,4,9-trithiobutane with trisulfide ligands, which could be used as an effective tripod surface anchor for Au NSPs [104]. They designed a hairpin-structured DNA probe containing a prostate-specific antigen (PSA)–nucleic acid aptamer sequence and attached it to the surface of Au NSPs. In the presence of PSA, the specifically recognized hairpin structure was disrupted. As the DNA probe on the surface of Au NSPs became less and less attached, the salt tolerance decreased significantly, so the aggregation produced a color change, based on which a sensitive colorimetric sensor was constructed.

Au NSPs can also be modified around macromolecules. As shown in Figure 9B, Ejeta et al. applied Au NSPs to the detection and quantification of the contaminant Cr^3+^ in water and modified them with 3-mercaptopropionic acid to improve the sensing recognition of metal ions in colorimetric detection [105]. A colorimetric assay of acrylamide was described by Shi et al. [106]. Acrylamide copolymerization could cause color changes due to increased distances among Au NSPs. In the presence of acrylamide, the copolymerization of Au NSPs with this substance increased the distance between Au NSPs, and the Au NSPs solution kept its original red color. 

Alizadeh et al. chose to modify Au NPs with sugar alcohols for the detection of Ag^+^ [72]. They introduced a colorimetric method that was highly selective for Ag^+^ and highly resistant to other metal ions. The assay was based on the measurement of plasmon resonance absorption changes on the surface of furfuryl alcohol-modified Au NSPs. Huang et al. described a colorimetric method for the determination of Al^3+^ with surface-modified Au NSPs [107]. They obtained Schiff bases using 2-hydroxy-1-naphthalaldehyde and 2-aminoethanol to functionalize Au NSPs by self-assembly. Complexation on Schiff bases of Au NSPs by Al^3+^ resulted in self-aggregation of Au NSPs accompanied by a color change from red to blue, which could be monitored by the naked eye or UV–vis absorption spectroscopy. 

### 3.3. Growth

**Figure 10:**
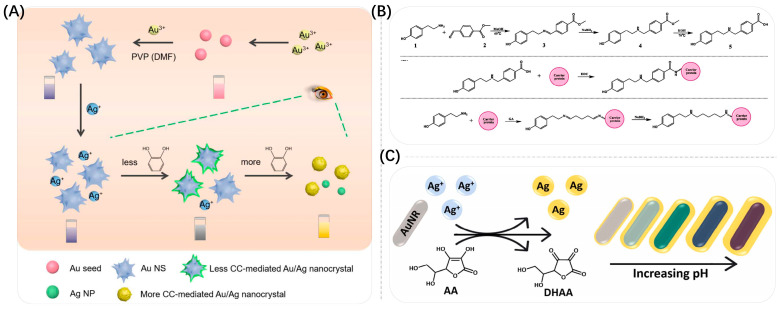
(**A**) Schematic illustration of the plasmonic colorimetric sensor for the detection of CC. Reproduced with permission from [74]. Copyright 2022, MDPI. (**B**) Synthetic routes of hapten Tyr-271; Scheme of coupling hapten Tyr-271 to carrier protein to form artificial antigen; Scheme of coupling hapten Tyr to carrier protein to form artificial antigen. Reproduced with permission from [113]. Copyright 2022, Elsevier. (**C**) Schematic illustration of the principle of the proposed multicolorimetric pH sensor. AA is oxidized to dehydroascorbic acid (DHAA) by silver ions. Reproduced with permission from [73]. Copyright 2022, Elsevier.

The growth of Au NPs refers to the generation of plasma nanoparticles or new plasma nanostructures by gold ions in the absence or presence of seeds, resulting in changes in the extinction spectrum of the LSPR and allowing a change in the color of the solution. Liu’s group reported the detection of proteases based on the in situ formation of Au NSPs. The protease substrate can be cleaved by the protease, leading to the exposure of the amino terminus of a fragment, which inhibits the catalytic oxidation of ascorbic acid (AA) by Cu^2+^ and, thus, promotes the formation of Au NSPs. This leads to an absorption peak at ∼530 nm and the solution turns bright red. The generated Au NSPs have a high extinction coefficient for quantitative and sensitive colorimetric analysis of proteases. The assay is capable of detecting proteases down to 1 nM with good recoveries and high specificity [108]. In the same way, Gopalakrishnan et al. developed a paper-based colorimetric assay for the determination of bilirubin based on the in situ reduction of chloroauric acid to form Au NPs [109]. The wax patterned paper strip was immobilized with chloroauric acid. In the presence of bilirubin, Au(III) was reduced and formed Au NPs. This led to a color change from yellow to purple. The detection limit was 1.0 mg/L. In contrast to Au NSPs, anisotropic nanostructures can exhibit multiple LSPR peaks, such as Au NRs with two LSPR bands that exhibit transverse and longitudinal resonance modes at shorter and longer wavelengths, respectively. The LSPR peaks can be easily adjusted by adjusting the aspect ratio of Au NRs, resulting in solutions with rich color variations. Compared with Au NRs, gold nano-bipyramids (Au NBPs) have two sharp ends, larger local electric field enhancement, larger extinction cross section and narrower longitudinal absorption peak width, which can make their color and spectral changes more obvious by means of etching or growth and, therefore, can improve the sensitivity of detection. In addition, gold nanostars (Au NSs) are widely used in the construction of biosensors due to their multiple branching structures, their sharp tips, large surface roughness and their strong LSPR properties [110]. The longitudinal LSPR peaks of Au NSs can be shifted from the visible region to the NIR region by expanding the branch length, increasing the amount of branches and nanoparticle size [111]. Branches and tips correspondingly control the arrangement of gold atoms to form high-refractive-index facets, resulting in high chemical and catalytic activity, which is potentially valuable for subsequent ultrasensitive sensor development. Usually, the growth of gold nanoparticles is achieved based on the reduction reaction of metal ions. Some targets can react directly with HAuCl_4_ and Ag^+^ based on their own reducing properties, or they can deposit Au or Ag on Au NPs’ surface by indirect reactions to generate strong reducing agents [112]. As shown in Figure 10A, Wang et al. proposed a plasmonic colorimetric sensing platform based on the reducing nature of the target Au NSs for the ultrasensitive detection of catechol (CC), where the target analyte CC was used to reduce Ag^+^ to form a silver coating on the surface of Au NSs, resulting in a blueshift in the LSPR of Au NSs [74]. With a gradual increase in CC concentration, the Ag coating on the surface gradually nucleated and the LSPR blueshift continued. The detection range of this strategy was from 3.33 nM to 107 μM with a detection limit of 1 nM, achieving an ultra-wide range and ultrasensitive CC detection, further facilitating the study of target-mediated LSPR sensors. 

As shown in Figure 10B, Luo et al. conducted work on enzymatic catalysis of the catabolism of non-reducing substrates into reduction products, which in turn indirectly mediated the growth of Au NSs [113]. They designed and synthesized a novel semi-antigen against tyramine (Tyr), generating a specific monoclonal antibody against Tyr and subsequently catalyzing the reaction of alkaline phosphatase (ALP) to produce AA, which in turn induced the growth of Au NSs. A colorimetric immunoassay for Tyr detection was developed and combined with smartphone-assisted signal readings, supported by a color recognizer application. The quantitative detection of Tyr was achieved by analyzing the red/blue channel values of the assay solution images taken by the smartphone. The solution produced a multicolor transition from pink to purple to blue in the presence of different concentrations of Tyr, which was more easily distinguished by the naked eye and smartphone. The detection limit for Tyr in beef, pork and yogurt was 19.7 mg/kg or L, providing a promising platform for field detection of Tyr. Similarly, Zhou et al. designed a colorimetric sensor for the detection of chloroacetamide herbicides based on the dephosphorylation of ascorbic acid 2-phosphate by ALP to produce AA that triggered the deposition of silver on Au NSs [114]. 

In addition, as shown in Figure 10C, Orouji et al. designed a colorimetric nanosensor for pH measurement using the reduction reaction between Au NRs, Ag^+^ and AA [73]. The strongly pH-dependent AA reduction of Ag^+^ led to the deposition of Ag nanoshells on the surface of Au NRs, forming Au@Ag core–shell nanorods that resulted in a series of blueshifts in their longitudinal peaks and distinct color changes from brownish pink/light green (acidic media) to dark green/blue (neutral media) and eventually purple/brown (alkaline media) depending on the pH which ranged from 2.0 to 12.0. And, by immobilizing Au NRs on the surface of filter paper, pH-sensitive paper-based analytical devices could be simply manufactured, ultimately enabling accurate pH monitoring of urine, spoiled meat, drinking water and seawater samples. Allen et al. used hollow Au NSPs (HGNs) as seeds to mediate the growth of multi-branched hollow Au NSs (HNSs) using AA to reduce Au^3+^ in the presence of Ag^+^ and achieved a widely tunable LSPR by controlling the length of the inner core and spikes [115]. 

### 3.4. Etching

By mediating the etching of Au NPs, the LSPR properties of Au NPs can be changed, which in turn changes the color of the reaction solution for colorimetric detection of targets. Further enhanced signal amplification linearly associated with analyte concentration is achieved. So far, Au NPs of different shapes, such as Au NSPs, Au NRs, Au NPLs, Au NBPs, Au NSs, etc., have been used as etching-based plasmonic colorimetric sensors for the detection of various target substances, such as DNA, proteins, metal ions, glucose and various small molecules. Sensing methods based on etching mechanisms are divided into the following categories: direct etching of the target, reaction formation complex-induced etching, oxidant-mediated etching and inhibitor-mediated etching (Table 2).

**Figure 11:**
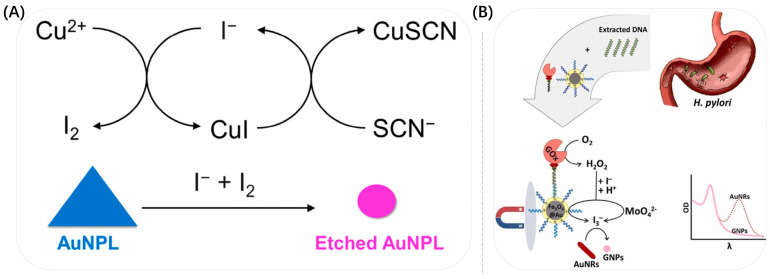
(**A**) Schematic Representation of the Proposed Mechanism for the Cu^2+^-Assisted I^−^-Mediated Au NPL etching. Reproduced with permission from [78]. Copyright 2017, ACS Publications. (**B**) The schematic illustrates the mechanism of the Au NR-etching-based sarcosine detection. Reproduced with permission from [116]. Copyright 2022, Elsevier.

The first approach to developing a colorimetric sensor based on the Au NP-etching mechanism is to use the target to directly etch Au NPs. Cu^2+^, I^−^ and Fe^3+^ plasmas have higher redox potentials than Au^+^ under certain conditions and can quickly and sensitively etch the high-energy surface of Au NP protrusions to tailor the surface plasmon resonance of Au NMs for colorimetric sensing of targets. As shown in Figure 11A, Mizuo Maeda et al. established a fast, simple and selective colorimetric method for the detection of Cu^2+^ using I^−^-mediated etching of triangular gold nanoplates (Au NPLs), which was accompanied by a drastic color change from blue to red [78]. Among them, Cu^2+^ acted as an oxidizing agent with I^−^ to generate I_2_, which etched Au NPLs, causing a color change in the solution. This system enabled the detection of Cu^2+^ on visual and spectroscopic analysis with detection limits of 10 μM and 1 μM, respectively. They further applied this Cu^2+^-assisted I^−^-mediated etching of Au NPLs to the aptamer detection of chloramphenicol (CAP). Based on the molecular recognition of DNA aptamers, the visual detection of CAP was achieved with a detection limit of 5 μM. Similarly, Zhong et al. used I oxidation into I_2_ to etch Au NRs along the longitudinal direction, resulting in a blueshift of the LSPR peak of Au NRs and visualized the detection of sarcosine by observing the color change of the solution [120]. As shown in Figure 11B, Kermanshahian et al. prepared a Au NR-etching-based H. pylori gene sensor with the advantages of good specificity, cost-effectiveness and high sensitivity [116]. Molybdate had HRP mimetic activity under acidic conditions, based on molybdate ions catalyzing the oxidation of I^−^ and accelerating I^−^-mediated etching of gold nanoparticles. Under optimal conditions, excellent sensitivity was obtained for H. pylori with a linear range and detection limit of 40–2040 aM and 31.8 aM, respectively.

**Figure 12:**
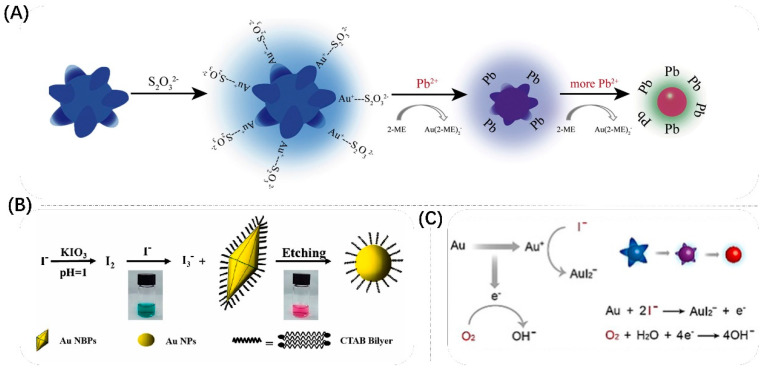
(**A**) Schematic diagram of Pb^2+^ detection by its catalytic activity after being reduced to Pb on etching GNSs in the presence of Na_2_S_2_O_3_ and 2-ME. Reproduced with permission from [117]. Copyright 2021, Elsevier. (**B**) Schematic diagram of colorimetric detection of iodine-etched Au NBPs. Reproduced with permission from [118]. Copyright 2023, Elsevier. (**C**) Illustration of the proposed mechanism and morphological change during the surface etching of Au NSs. Reproduced with permission from [121]. Copyright 2021, Wiley.

In general, Au is highly stable and not easily etched by oxidation, so researchers used Pb^2+^, Hg^+^, I^−^, Cl^−^ and CN^−^ plasma to form complexes with Au NMs to make them redox to form complexes, thus achieving etching of Au NMs. As shown in Figure 12A, Cao’s group proposed an extremely sensitive detection method for Pb^2+^ [117]. They used Au NSs as probes and covered them with sodium citrate. After further addition of Pb^2+^ and 2-mercaptoethanol (2-ME), Pb^2+^ was reduced to Pb and deposited on the particles’ surface, which accelerated the etching of Au NSs by 2-ME and generated Au(2-ME)^2−^. They used 4-(2-hydroxyethyl)-1 piperazineethanesulfonic acid as a reducing and capping agent to prepare Au NSs for the quantification of Pb^2+^ with high stability and sensitivity. The color of the solution changed from blue-green to blue, to purple to red and, finally, to colorless when the Pb^2+^ concentration was increased to the range of 0–10 μM. Even when the Pb^2+^ concentration was as low as 200 pM, the color change could be distinguished with the naked eye. Pb^2+^ could still be detected by monitoring the longitudinal LSPR peaks of Au NSs with a detection limit of 1.5 pM and an operating range of 2 pM-1 μM. As shown in Figure 12B, Liu et al. developed a colorimetric method for the rapid determination of I^−^ in kelp based on the fact that under acidic conditions, IO_3_^−^ could react rapidly with I^−^ in kelp to form I_2_ and, subsequently, I_2_ reacted with I^−^ to form the intermediate I_3_^−^ [118]. In the presence of CTAB, Au NBPs were corroded by I_2_ from I_3_^−^ to produce AuI_2_^−^(CTA^+^)_2_, resulting in a decrease in the aspect ratio of Au NBPs, forming a significant blueshift of the longitudinal peak of LSPR and a color change of the colloid from blue-green to red. In the range of 4.0 μM to 15.0 μM, a linear relationship between the change in LSPR peak and I^−^ concentration could be found, with a sensitive detection limit of 0.2 μM for UV–vis spectrometry and a concentration of 4.0 μM when a significant color change was observed by the naked eye. As shown in Figure 12C, Yunlei et al. developed a rapid and sensitive surface-etching mechanism of iodide-induced Au NSs that not only changed the color of the solution from blue to purple and, finally, to red in 20 min for fast instrument-free readout of iodide detection but also had a high sensitivity to distinguish concentration differences of 20 nM [121]. Au NSs exhibit polarization-dependent scattering and absorption with multiple spectral peaks and are particularly susceptible to changes in the eccentricity of prominent tips. They characterized the effect of different concentrations of iodide on the Au NSs’ morphology by transmission electron microscopy. An amount of 1 μM iodide caused Au NSs to become less branched nanostructures with rounded tips, while Au NSs under 10 μM iodide incubation were etched into spherical nanostructures. The surface etching of Au NSs was achieved by using energy-dispersive X-ray spectroscopy measurements, which revealed that iodide bound to the surface of Au NS tips to form the complex AuI_2_^−^. Based on the above principles, Hong et al. developed a colorimetric method based on I^−^-mediated etching of Au NSs for the highly sensitive determination of nitrite with a detection limit of 0.4 μM [122]. 

**Figure 13:**
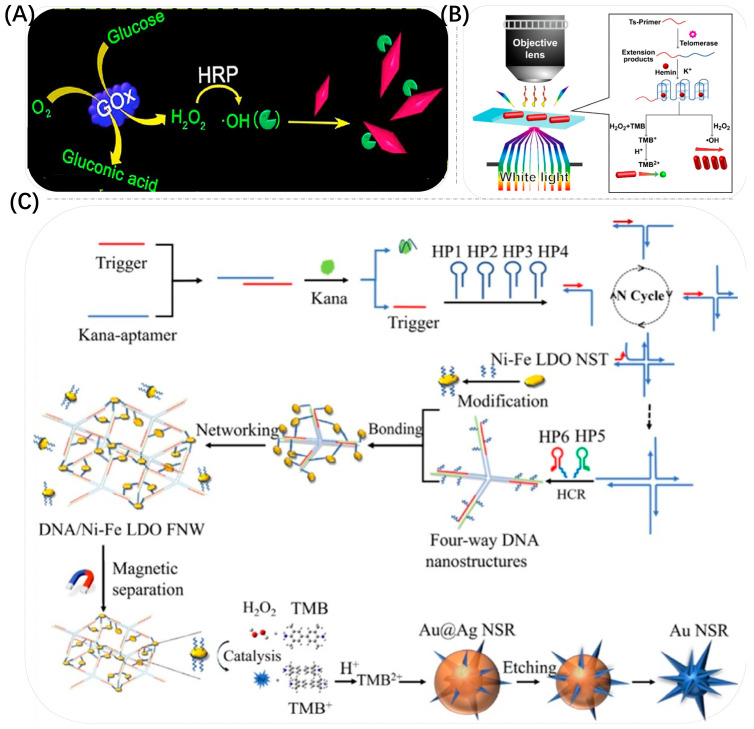
(**A**) Schematic illustration of the Au NBP-etching-based multicolor colorimetric assay for glucose detection. Reproduced with permission from [77]. Copyright 2019, Elsevier. (**B**) Schematic of LSPR DFM Scattering Detection of Telomerase Activity via TMB^2+^ Etching of Au NRs at a Single-Au NR Level. Reproduced with permission from [124]. Copyright 2022, ACS Publications. (**C**) Scheme of the determination of Kana based on the etching of Au@Ag NSR by DNA/LDO FNW. Reproduced with permission from [76]. Copyright 2021, Elsevier.

A third approach to developing a colorimetric sensor based on the etching mechanism of Au NPs is to oxidatively etch Au NSPs using Co^2+^ and various enzymes to generate strong oxidants such as ·OH, superoxide radicals (O_2_^−^·) and H_2_O_2_. As shown in Figure 13A, Xu et al. proposed ultrasensitive multicolor colorimetric detection of blood glucose based on the enzyme-catalyzed reaction-mediated etching of Au NBPs with fast analysis speed, high specificity, no need for fine and complex detection equipment, and a simple operation process [77]. Under the catalytic effect of HRP, the H_2_O_2_ generated by the oxidation of glucose decomposes into the strongly oxidizing ·OH, which could accelerate the etching of Au NBPs. Different levels of concentration of glucose caused different degrees of etching, resulting in a blueshift in the LSPR bands of Au NBPs, accompanied by various color changes recognizable to the naked eye (e.g., light brown, blue and pink, etc.). Since Au NBPs had two sharper vertices, the aspect ratio could be changed more easily, which greatly improved the detection sensitivity. The proposed colorimetric glucose detection method on this basis achieved a dynamic range of 0.05–90 μM with a detection limit of 0.02 μM. Deng et al. also achieved controlled etching of Au NBPs based on an enzymatic reaction catalyzing the production of ·OH from H_2_O_2_, resulting in a change in solution color and detection of carbaryl with a detection limit of 0.26 mg/kg and the ability to capture visual images using a smartphone [123]. As shown in Figure 13B, Yang et al. developed a fast and sensitive telomerase activity assay based on TMB^2+^ etching Au NRs at the single-particle level [124]. The telomerase primer is triggered by telomerase to extend to form heme/G-quadruplex DNAase, which in turn catalyzed the production of ·OH to oxidize TMB to TMB^2+^, further etching Au NRs efficiently, resulting in changes in the LSPR of Au NRs and multiple color changes in the solution. Compared with the common strategy of direct etching of Au NRs by ·OH, higher stability of TMB^2+^ greatly overcame the disadvantage of the short lifetime of ·OH to effectively etch Au NRs. As shown in Figure 13C, Yao et al. also used TMB^2+^ to oxidatively etch Ag @Au NSs to expose the tips of Au NSs, resulting in significant changes in the LSPR signal [76]. The LSPR exhibited a redshift in UV–vis absorption and achieved extremely low detection limits through quadruple amplification. The sensitivity of the system was significantly improved by introducing a novel concept of “cusp-exposure”. The practical applicability of this strategy was successfully demonstrated by the determination of Kana in real human milk and urine samples under optimal experimental conditions with a detection limit of 3 aM, a signal-to-noise ratio of 3 and a calibration curve linearity range of 0.01 fM–0.1 nM. He et al. constructed an ultrasensitive multicolor colorimetric sensor based on catalytic oxidation reactions for the detection of Fe^2+^ by etching Au NBPs [125]. Firstly, they prepared Au NBPs using seed-mediated growth methods and produced them with good homogeneity using a three-step purification process. The Au NBPs were rapidly etched by the O_2_^−^· generated by the reaction of Fe^2+^ and H_2_O_2_ along the longitudinal direction, resulting in a blueshift of the LSPR and a significant color change. Under optimal reaction conditions, the peak shift of Au NBPs was linear in the logarithm of Fe^2+^ concentration in the range of 10 nM–10 μM with a detection limit of 1.29 nM. Zhou et al. proposed an effective, selective and multicolor colorimetric detection method for Cu^2+^ based on the modulation of peroxidase-like nano-enzyme-mediated etching of Au NRs [75]. The Cu^2+^–sarcosine complex was chosen as a nano-enzyme that could catalyze the generation of TMB^2+^ from TMB in the presence of H_2_O_2_, etching Au NRs, and then achieving the determination of Cu^2+^ based on the blueshift of the longitudinal LSPR peak of Au NRs. Under optimal conditions, the colorimetric detection method had a high sensitivity with a linear range of 0.05–4.0 μM at a detection limit of 0.034 μM for Cu^2+^. With an increase in Cu^2+^ concentration, the solution exhibited a rainbow-like color, which allowed qualitative analysis of Cu^2+^ detection by the naked eye. Wu et al. developed an effective optical sensing array and used it for the identification of multiple antioxidants based on the etching of two different morphologies of Au NMs by the oxidation products of TMB [126]. Different targets triggered different degrees of etching of Au NMs, resulting in changes in wavelength and color of the solution. The discrimination and determination of eight antioxidants were achieved by fingerprinting, linear discriminant analysis (LDA) and hierarchical cluster analysis (HCA) plots with a detection limit of 100 nM. 

A final sensing strategy based on the Au NP-etching mechanism is the use of inhibitors to mediate etching. The target can keep the shape of the LSPR unchanged by hindering the etching of Au NPs and the etching rate can be varied by controlling the concentration of the inhibitor under specific conditions. Luo et al. developed a multicolor biosensor for ALP activity detection based on the peroxidase-like activity of Cu NCs and the etching of Au NRs [119]. Cu NCs could catalyze the oxidation of TMB to generate TMB^2+^, which in turn etched Au NRs, and the etching process was accompanied by a significant change in the color of the system. The presence of sodium pyrophosphate (PPi) inhibited the peroxidase-like activity of Cu NCs, which could be restored by the addition of ALP. The addition of different concentrations of ALP led to the restoration of the catalytic activity of Cu NCs to different degrees and generated different amounts of TMB^2+^, which affected the morphology of Au NRs in the system and made the output color signal change. Moreover, the reaction process did not require any complex instrumentation. The biosensor showed a linear relationship with ALP activity in the range of 10.0~80.0 UL^−1^ with a detection limit of 4.6 UL^−1^. Similarly, Qin et al. established an enzyme-free colorimetric method for the sensitive detection of UA by inhibiting the etching of Au NRs [7]. Since the reaction of manganese dioxide nanosheets (MnO_2_ NS) and I^−^ produced the strong oxidant I_2_, which could etch Au NRs, the introduction of UA rapidly reduced MnO_2_ NS to Mn^2+^, which reduced the formation of I_2_ and inhibited the etching of Au NRs. Under optimized conditions, the sensitive detection range of UA was 0.8–30 μM and 30–300 μM, and the detection limit in the low-concentration range was 0.76 μM.

## 4. Conclusions

Au NPs, as popular nanomaterials, have been used as building blocks for colorimetric sensors by many researchers because of their high stability, good biocompatibility and ease of modification. In this review, we have discussed the research progress on colorimetric sensing of Au NPs based on enzyme-like activity and LSPR of Au NPs dependent on size, morphology and functionalization, including Au NSPs, Au NRs, Au NPLs, Au NBPs, Au NSs and some hybrids in which Au and other substances function together. The review shows that colorimetric sensors based on catalytic sensing and LSPR wavelength shift sensing of Au NPs can easily detect various chemically and biologically relevant molecules, such as those related to ion detection, biomarkers, glucose detection, protein detection, etc. Among them, gold nano-enzymes can exhibit enzyme-like activities and have physicochemical properties superior to those of natural enzymes. Numerous studies have shown that hybridized Au NPs mixed with multiple components exhibit better catalytic properties in terms of bioactivity. In addition, shape-controlled colorimetric sensors constructed using the LSPR properties of Au NPs are more promising than sensors of gold nano-enzymes catalyzing a single chromogenic substrate. Compared with traditional detection methods, colorimetric sensors developed based on shape-controlled Au NPs can achieve multicolor and efficient detection purposes. These sensors are not only easy to operate and have low detection cost, but are also able to respond quickly to the detection of the target. Combining the enzyme-like activity, the LSPR properties and other different physicochemical properties of Au NPs, as well as using enzymatic research theories and methods to study and design Au NMs, will not only make it easier to understand their catalytic behaviors in biological systems, but also facilitates the promotion of related applications of colorimetric sensing of biomolecules based on Au NPs. However, despite the many advantages of Au NPs in colorimetric detection, they still face the following limitations: (1) The catalytic activity of gold nano-enzymes may be affected by various factors in the biological environment, such as pH and temperature, which reduces their catalytic efficiency. (2) The detection sensitivity and specificity of Au NPs still need to be improved. (3) Since the size and shape of Au NPs are not precisely controlled, they are still in the experimental stage and cannot be industrially produced. In the future, with the development of nanotechnology, it is necessary to further develop novel colorimetric sensors based on Au NPs to overcome the limitations due to the complexity of the detection environment and samples and to realize the visual detection of multiple targets, high stability, high sensitivity and high specificity.

## Figures and Tables

**Table 1:** LSPR-based applications of Au NPs with different modification methods and shapes.

Modification Method	Type of Au NPs	Type of Target	Time of Color Change	The Limit of Detection	Reference
Aggregation	Au NSPs	Gram-positive and Gram-negative strains	Within 20 min	10^4^ CFU/mL	[69]
Aggregation	Au NSPs	Tetracycline and chloramphenicol	16 min	32.9 nM & 7.0 nM	[70]
Aggregation	Au NSPs	Tl	2 min	3.2 nM	[71]
Surface modification	Au NSPs	Ag(I)	10 min	12 nM	[72]
Growth	Au NRs	pH	3 min	pH 2.0 ~ 12.0	[73]
Growth	Au NSs	Catechol	2 h	1 nM	[74]
Etching	Au NRs	Cu^2+^	15 min	0.034 μM	[75]
Etching	Au NSs	Kanamycin	3 min	3 aM	[76]
Etching	Au NBPs	Blood glucose	30 min	0.02 mM	[77]
Etching	Au NPLs	Cu^2+^ and chloramphenicol	20 min	10 uM & 5 uM	[78]
Etching	Au NSPs	*S. aureus*	65 min	10 CFU/mL	[79]

**Table 2:** Sensing methods based on etching.

Ways Based on Etching	Type of Au NPs	Targets	Color Change	The Limit of Detection	Reference
direct etching of the target	Au NPLs	Chloramphenicol	from blue to red	10 μM & 1 μM	[78]
direct etching of the target	Au NRs	Helicobacter pylori	from blue to purple to red	40–2040 aM & 31.8 aM	[116]
reaction formation complex-induced etching	Au NSs	Pb^2+^	from blue-green to blue, to purple to red, and finally to colorless.	1.5 pM	[117]
reaction formation complex-induced etching	Au NBPs	I^−^	from blue-green to red	4 μM & 0.2 μM	[118]
oxidant-mediated etching	Au NBPs	Glucose	from light brown to blue to pink	0.02 μM	[77]
oxidant-mediated etching	Au NRs	Cu^2+^	rainbow colors	0.034 μM	[75]
inhibitor-mediated etching	Au NRs	ALP	from light brown to green to blue to pink	4.6 UL^−1^	[119]
inhibitor-mediated etching	Au NRs	UA	from light brown to blue to pink	0.76 μM	[7]

## Data Availability

Not applicable.
